# A classical algorithm to mimic quantum decisions

**DOI:** 10.1093/nsr/nwz081

**Published:** 2019-07-25

**Authors:** Mile Gu

**Affiliations:** School of Physical and Mathematical Sciences, Nanyang Technological University, Singapore

In his famous 1982 lecture, Nobel laureate Richard Feynman seeded the idea of quantum computation. He reasoned that the amount of classical information required to describe a quantum system scaled exponentially with its size, and thus could not be efficiently simulated by a classical computer. Indeed, a fairly modest system of 100 quantum bits would already require more information to describe than all the present data storage on Earth [1]. Given this, there would likely be computational tasks that achieve *quantum supremacy*—impossible to perform practically without the aid of a quantum information processor. In the 37 intervening years, these initial ideas have solidified, as the discovery of a string of quantum algorithms—from efficient factoring of large numbers to simulating chemical reactions—fueled a race for quantum supremacy.

Yet, even theoretically, conclusively deciding—if quantum supremacy has been achieved—is non-trivial. Judging what problems can be efficiently solved by classical computers turns out to be exceedingly hard in itself. Take factoring, for instance. It is really classical intractable, or is it merely that we have not yet found an efficient classical means to factor? As we better understood how quantum computers process information, we also got better at finding classical methods to simulate them. This in turn has inspired new efficient classical algorithms in surprising situations [2].

Prof. Man-Hong Yung, associate professor at the Southern University of Science and Technology, and colleagues provided a beautiful illustration of such a discovery. Their work has been published in the July 2019 issue of *National Science Review* [3]. It considers a specialized type of linear optical quantum processor. The input and output are encoded within an array of optical modes by adjusting their initial photon number. Computation is achieved by interfering with these modes via a network of beam splitters, such that photons in one mode are transferred to another.

Such processors have captured scientific attention due to their ability to perform a task known as boson sampling. When each mode is initialized with a single photon, the output statistics allows efficient evaluation of the matrix permanent—a computational problem with very strong evidence for being classically intractable [4]. This, coupled with their relative ease of engineering, made this specialized type of quantum processor an ideal holy grail for demonstrating quantum supremacy.

Given this, one may expect that classical computers can say little about what such processors will output. After all, the number of paths that a photon can take to reach one mode from another scales exponentially with the size of the beam-splitter network. One may therefore assume that it would take exponential resources to track these paths to determine the probability of a photon moving from mode A to mode B. However, Yung and colleagues showed that a classical algorithm can efficiently estimate the transition probabilities—as long as one allows for additive error. This result represents a significant generalization of prior results by Scott Aaronson, which considers the case where there exists at most one photon in each mode [5]. In contrast, Yung's solution works regardless of the number of photons encoded in each mode.

The result not only benefits the simulation of bosonic interference, but also has strong consequences in computing. Suppose that we use such specialized quantum processors to make binary decisions. That is, depending on input, we feed in particular distributions of photons—and then decide to take a particular action depending on whether the probability of seeing *x* photons in a particular output is above some threshold. In such contexts, a classical algorithm that estimates this probability up to the bounded additive error is sufficient for making identical decisions as the quantum processer itself. As such, Yung's algorithm enables a classical computer to be able to efficiently mimic the decisions of its specialized linear optical counterpart. This will no doubt have important ramifications in the present race for quantum supremacy. Much present effort is spent on demonstrating quantum supremacy through boson sampling, and the results here indicate that we will need to introduce more sophisticated quantum architectures if we wish to demonstrate such supremacy in making the right decisions.

## References

[1] Reinsel D , Gantz J. IDC White Paper, #US44413318, 2018.

[2] Tang E . arXiv:1807.04271.

[3] Yung M-H , Gao X, Huh J. Natl Sci Rev 2019; doi:10.1093/nsr/nwz048. PMC8291458 34691927

[4] Aaronson S , Arkhipov A. Annual ACM Symposium on Theory of Computing 2011; 333–42.

[5] Aaronson S , Hance T. Quantum Inf Comput 2012; 14: 541–59.

